# Comparison of the effects of different percentages of soy protein in the diet on patients with type 2 diabetic nephropathy: systematic reviews and network meta-analysis

**DOI:** 10.3389/fnut.2023.1184337

**Published:** 2023-08-24

**Authors:** Jun Sun, Yu Wei, Runyu Miao, Xiangyuan Zhang, Boxun Zhang, Lili Zhang, Linhua Zhao

**Affiliations:** ^1^Affiliated Hospital of Changchun University of Traditional Chinese Medicine, Changchun, China; ^2^Institute of Metabolic Diseases, Guang’anmen Hospital, China Academy of Chinese Medical Sciences, Beijing, China; ^3^Graduate College, Beijing University of Traditional Chinese Medicine, Beijing, China

**Keywords:** soy protein, percentages, type 2 diabetic nephropathy, systematic reviews, network meta-analysis

## Abstract

**Background:**

Dietary soy protein (SP) is a potential intervention for protecting the kidneys and improving glucose and lipid metabolism. However, whether this effect is related to the percentage of SP intake remains unclear.

**Objective:**

This study aims to review and analyze the results of randomized clinical trials (RCTs) in patients with type 2 diabetic nephropathy (T2DN) who received diets with different percentages of SP.

**Methods:**

The databases: PubMed, Embase, Cochrane Central Register of Controlled Trials (CENTRAL), Web of Science, China National Knowledge Infrastructure (CNKI), Chinese BioMedical Literature Database (CBM), WanFang, Weipu (VIP), and ClinicalTrials.gov were searched until February 2023, for RCTs on T2DN and SP.

**Results:**

A total of six studies comprising 116 participants were included. The interventions were classified as 0% SP, 35% SP, and 100% SP. To improve serum creatinine (Scr), blood urea nitrogen (BUN), 24-h urine total protein (24hUTP), and glomerular filtration rate (GFR), a 35% SP diet was the most effective, compared to a 0% SP diet, which showed a mean difference of −154.00 (95% confidence interval: −266.69, −41.31) for 24hUTP. Although it had significant benefits for 24hUTP, great heterogeneity was observed. To improve the glycolipid metabolism-related markers such as cholesterol (CHO), high-density lipoprotein cholesterol (HDL-C), low-density lipoprotein cholesterol (LDL-C), fasting blood glucose (FPG), and weight, the 35% SP diet demonstrated superior efficacy compared to the 0% SP diet. Specifically, the mean difference for CHO was −0.55 (95% confidence interval: −1.08, −0.03), and for LDL-C, it was −17.71 (95% confidence interval: −39.67, −4.24). The other indicators were not statistically significant. Most studies had concerns regarding the risk of bias.

**Conclusion:**

The findings of this study demonstrate that both 35% and 100% SP diets are more effective than a diet with no SP in improving renal function and glucolipid metabolism in patients with T2DN. As a result, a diet incorporating 35% SP may be the optimal choice for individuals with T2DN.

**Systematic review registration:**

https://www.crd.york.ac.uk/PROSPERO/display_record.php?RecordID=352638, identifier CRD42022352638.

## 1. Introduction

The latest global diabetes map (1) released by the International Diabetes Federation (IDF) in 2021 revealed that the number of adult patients with diabetes (20–79 years old) worldwide has reached 537 million. Diabetic nephropathy (DN) is the most common microvascular complication in patients with type 2 diabetes (2). Chronic hyperglycemia leads to the dysfunction of various cell types in the kidney and eventually leads to progressive renal failure (3). Traditionally, DN is diagnosed based on persistent hyperalbuminuria and a subsequent decline in the glomerular filtration rate (GFR) (4). More than 40% of patients with diabetes eventually develop DN, and a considerable number develop kidney failure, requiring dialysis or transplantation (4, 5). In developed countries, DN accounts for 44.5% of end-stage renal disease (ESRD) (6), resulting in a public health burden.

Patients with DN are recommended for comprehensive management, usually by choosing diet therapy, glycemic control, blood pressure control with renin-angiotensin system (RAS) inhibitors, and lipid control with statins to inhibit DN progression (7–9). In addition, dietary protein intake has attracted the interest of researchers worldwide. Brenner et al. (10) hypothesized that excess protein intake contributes to the progressive deterioration of renal function in the diabetic state. Therefore, limiting protein intake can reduce hyperfiltration in patients with diabetes. Anderson et al. (11) proposed a new hypothesis. They suggested that substituting soy protein (SP) with animal protein in patients with diabetes would lead to less hyperfiltration and glomerular hypertension, thus preventing the occurrence and progression of DN. This hypothesis has been confirmed in a recent prospective study. The investigators suggested that the effect of protein consumption on the risk of end-stage renal failure might depend on the type of protein source. Red meat consumption was strongly associated with ESRD risk in a dose-dependent manner, whereas other proteins, such as fish, did not show such deleterious associations (12). It is worth noting that there was a negative correlation between the consumption of soybean and beans and ESRD risk. However, there is no clear evidence to clarify the relationship between the percentage of SP intake in the diet and the prognosis of patients with type 2 diabetic nephropathy (T2DN).

**FIGURE 1:**
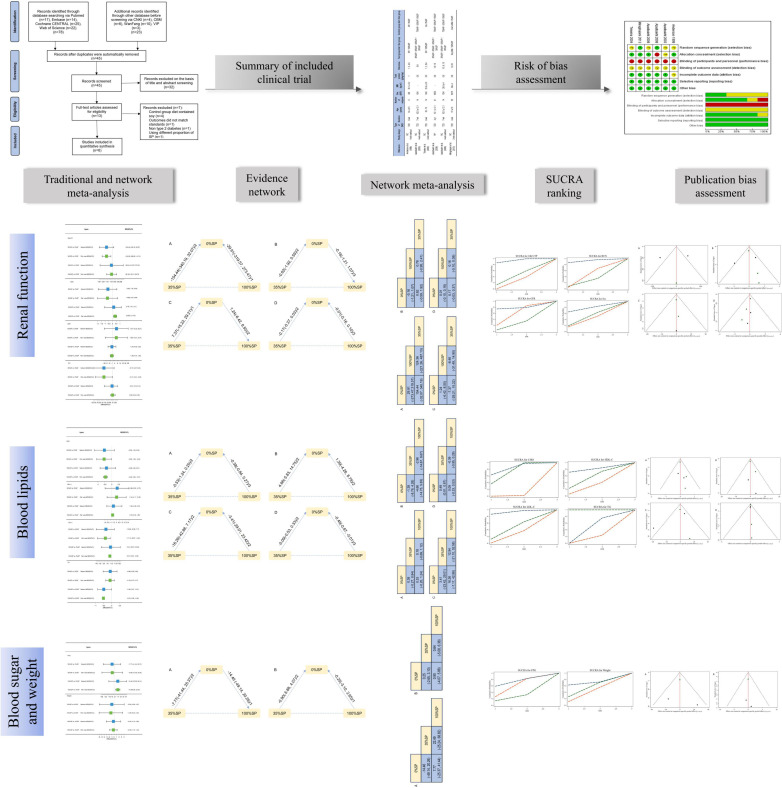
Flowchart of the study.

Therefore, we conducted a network meta-analysis of randomized clinical trials (RCTs) on SP intake in patients with T2DN and evaluated the effects of different percentages of SP in the diet on primary renal outcome measures and markers related to glucose and lipid metabolism in patients with T2DN. Figure 1 presents a flowchart of the research method.

## 2. Materials and methods

This study was reported according to the PRISMA Extension Statement for Reporting Systematic Reviews Incorporating Network Meta-analyses (13). The International Prospective Register of Systematic Reviews (PROSPERO) registration number is CRD42022352638.

### 2.1. Literature search strategy

We systematically searched seven electronic databases, including PubMed, Embase, the Cochrane Central Register of Controlled Trials (CENTRAL), Web of Science, China National Knowledge Infrastructure (CNKI), Chinese BioMedical Literature Database (CBM), WanFang, and VIP, for randomized clinical trials investigating SP intake in patients with T2DN. The retrieval period ranged from the earliest record to February 2023. Our retrieval used Medical Subject Headings (MeSH) and text words from PubMed. In addition, we also searched “DN” related studies in the ClinicalTrials.gov database to find clinical trials related to SP. Supplementary Table 1 provides a detailed description of the search strategies used.

### 2.2. Inclusion and exclusion criteria

Studies that met the following criteria were included: (1) Participants: T2DN patients aged ≥18 years; (2) Intervention Group: Individuals with a diet containing SP; (3) Control Group: Subjects who were given a placebo or diet without SP; (4) Outcome Measures: The study reports included at least one of the kidney-related indicators: serum creatinine (Scr), blood urea nitrogen (BUN), 24-h urine total protein (24hUTP), glomerular filtration rate (GFR), or related indicators of glucose and lipid metabolism such as cholesterol (CHO) and high-density lipoprotein cholesterol (HDL-C), triglycerides (TG), low-density lipoprotein cholesterol (LDL-C), fasting blood glucose (FPG). (5) Study Design: only randomized controlled clinical study; (6) Publication Source: Articles or letters published in peer-reviewed journals with no restrictions based on the language of publication.

The exclusion criteria were as follows: (1) repeatedly published articles, incomplete data, reviews, animal studies, or *in vitro* cell experiments; (2) Studies focusing on renal failure, dialysis, and kidney transplantation; (3) Studies whose primary objective did not involve observing the effects of SP.

### 2.3. Literature screening and data extraction

The retrieved articles were imported into the Endnote version 20 Software. After eliminating duplicate literature, two researchers (J.S. and RY-M) were assigned to preliminarily screen the retrieved articles by reviewing the titles and abstracts. The full texts of the selected studies were obtained and further filtered according to the inclusion and exclusion criteria.

The extracted data included the following information: title, author(s), study design, duration, age, sex, number of people, body mass index (BMI), total protein, diet composition of the experimental and control groups, renal function-related indicators (Scr, BUN, 24hUTP, GFR), glucose and lipid metabolism-related indicators (FPG, CHO, TG, HDL-C, and LDL-C), and weight. Data extraction also adopted independent double-entry and crosschecking.

### 2.4. Risk of bias assessment

The quality of the included studies was evaluated according to the risk-of-bias tool from the Cochrane group (14), including bias sources such as random sequence generation, allocation hiding, blinding (divided into investigator/patient blinding and results from evaluator blinding), data integrity, and selective reporting of research results. Bias risk was divided into “low risk,” “high risk,” and “uncertain risk.” Review Manager (RevMan) version 5.4.1 software was used to summarize the results of the bias risk assessment.

### 2.5. Statistical analysis

We conducted two meta-analyses. First, we used the Revman 5.4.1 software to run a pairwise meta-analysis. I^2^ Statistical data were used to assess heterogeneity. If I^2^ < 50%, the fixed effects model was used; if I^2^ > 50%, the random effects model was used. In addition, we conducted a sensitivity analysis of indicators with heterogeneity. The results are shown in Supplementary Figure 1. These indicators were manually checked to analyze the possible causes of heterogeneity. We used the ADDIS software based on the Bayesian framework for network element analysis. In addition, the mean difference (MD) and 95% confidence interval (CI) were used as outcome indicators to express the pairwise and network meta-analysis results. The evidence network of different intervention measures was summarized using a network structure chart, and a league table was used to compare the efficacy of any two treatment methods. Stata version 15.0 (Stata Corp., College Station, TX, USA) was used to calculate the area under the cumulative ranking curve (SUCRA). The higher the SUCRA value, the better the intervention effect. A calibration comparison funnel chart was used to evaluate whether the small-sample effect caused bias in the intervention network.

## 3. Results

### 3.1. Process and information for included studies

**FIGURE 2:**
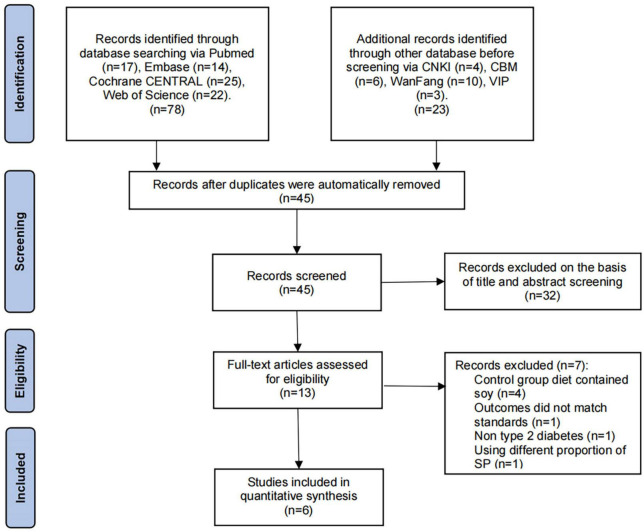
Test search and selection process.

A detailed flow of the study’s retrieval and screening process is illustrated in Figure 2. A total of 17 articles were retrieved from PubMed, 14 from Embase, 25 from Cochrane Central, 22 from Web of Science, 4 from CNKI, 6 from CBM, 10 from Wanfang, 3 from VIP, and 2 from ClinicalTrials.gov. After the results found by the above methods were merged and deduplicated, 45 articles remained. Of these, 32 were excluded based on title and abstract screening. Finally, 6 of the 13 articles that underwent complete text evaluation met the inclusion criteria, including five randomized crossover trials and one randomized parallel trial. A total of 116 patients were included in six studies, and the impact of seven diets with different protein compositions on the patients was evaluated. We divided the patients into three groups according to the amount of SP in the diet for the network meta-analysis. There were no statistically significant differences in the baseline data in any of the studies. Table 1 presents the grouping and experimental design. See Supplementary Table 2 for the results and data included in this study.

**TABLE 1:** Summary of included clinical trial.

References	Study design	Type of DN	Duration	Age (years)	Number of subjects	Sex (M/F)	BMI (kg/m^2^)	Total protein (g/kg/day)	Outcomes	Text group diet/Our group	Control group diet/Our group
Anderson et al. (11)	RC 4-week washout	T2D	8-week	64 ± 6.5	8	8/0	35.1 ± 6.2	1	1, 2, 4–6	SP/100%SP	AP/0%SP
Azadbakht et al. (27)	RC 4-week washout	T2D	7-week	62.5 ± 12.1	14	10/4	–	0.8	5–8	35%SP+35%AP+ 30%VP/35%SP	70%AP+30% VP/0%SP
Teixeira et al. (28)	RC 4-week washout	T2D	8-week	53–73	14	14/0	29.8 ± 0.8	0.5	1, 2, 5–8	SP/100%SP	Ca/0%SP
Azadbakht et al. (29)	RP	T2D	4-year	62.1 ± 12.1	41	18/23	–	0.8	1–9, 10	35%SP+35%AP+ 30%VP/35%SP	70%AP+ 30%VP/0%SP
Azadbakht and Esmaillzadeh (30)	RC 4-week washout	T2D	7-week	62.5 ± 12.1	14	10/4	26.6 ± 4	0.8	1–3, 9, 10	35%SP+35%AP+ 30%VP/35%SP	70%AP+30% VP/0%SP
Miraghajani et al. (24)	RC 2-week washout	T2D	4-week	51 ± 10	25	10/15	28 ± 4	0.8	1–8, 10	Soy Milk/100%SP	Cow’s Milk/0%SP

AP, animal-protein; DN, diabetic nephropathy; M/F, number of male and female; RC, randomized crossover design; RCT, randomized controlled trial; RP, randomized parallel design; SP, soy-protein; T2D, type 2 diabetic; VP, vegetable-protein. Outcomes: 1. Scr. 2. BUN. 3. 24hUTP. 4. GFR. 5. CHO. 6. TG. 7. HDL-C. 8. LDL-C. 9. FPG. 10. Weight.

### 3.2. Risk of bias assessment

**FIGURE 3:**
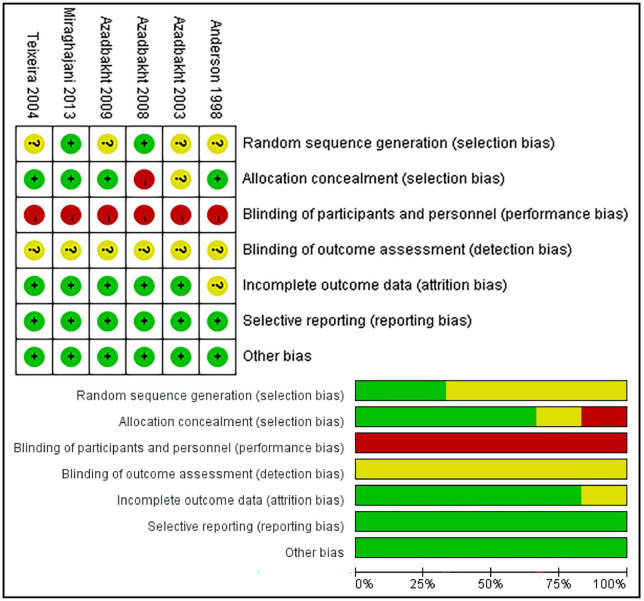
Risk of bias summary and risk of bias graph. Green, low risk; yellow, unclear; red, high risk.

According to the standard methods recommended by the Cochrane Collaboration for assessing death risk, three of the six ion studies showed clear random sequence genera. Four studies reported allocation concealment and none of the studies noted the blinding of participants and personnel. In addition, none of the studies mentioned the blinding of the outcome assessment and five studies had complete outcomes data. Owing to the small number of original studies, all studies were included in the analysis without considering the bias score. The risk assessment of bias is illustrated in Figure 3.

### 3.3. Effects of different percentages of soy protein on renal function

#### 3.3.1. Traditional pairwise and network meta-analyses

**FIGURE 4:**
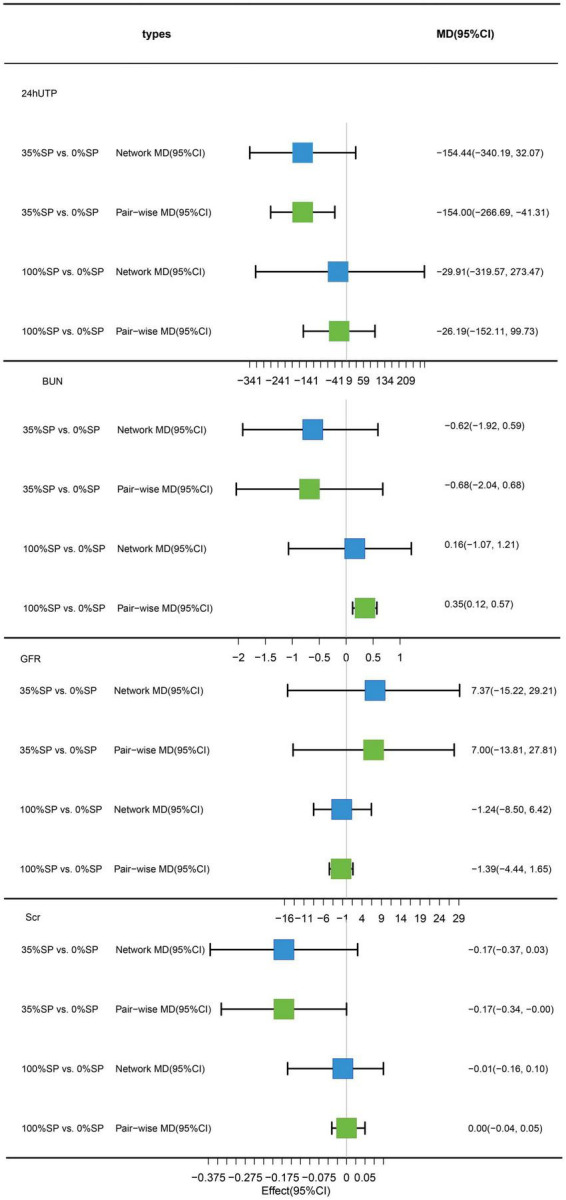
Network meta-analysis results (blue panels) and traditional pairwise meta-analysis results (green panels) show results for 24-h urine total protein (24hUTP), blood urea nitrogen (BUN), glomerular filtration rate (GFR), and serum creatinine (Scr). The line with the 95% CI represents the 95% CI in the network and traditional pairwise meta-analyses. When traditional meta-analyses are not feasible in specific comparisons, the results of a single trial can serve as direct evidence.

Traditional pairwise and network meta-analyses were performed on these four indicators. The results showed that the efficacy of the 35% SP diet was higher than that of the 0% SP diet, and the result of 24hUTP was statistically significant; 35% SP vs. 0% SP diet had an MD of −154.00 (95% CI: −266.69, −41.31) for 24hUTP (Figure 4).

#### 3.3.2. Network meta-analysis

**FIGURE 5:**
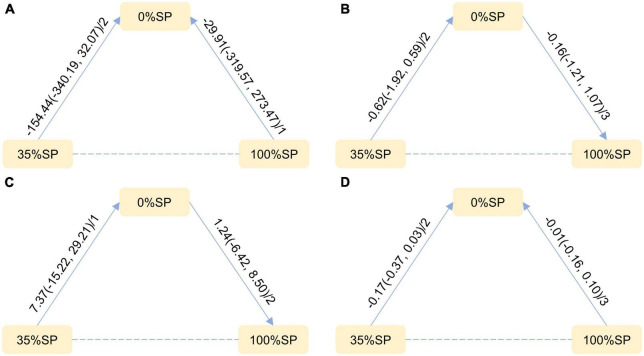
Network Evidence Map of Kidney Indicators: **(A)** 24-h urine total protein (24hUTP), **(B)** Blood urea nitrogen (BUN), **(C)** Glomerular filtration rate (GFR), **(D)** Serum creatinine (Scr). For pairwise comparisons in each indicator, arrows point to the less effective diet group, solid lines represent direct comparisons, and dashed lines represent indirect comparisons. Results for direct comparisons are network random effects meta-analyses, expressed as MD (95% CI)/number of trials. Results from a single trial were provided when the meta-analysis was not feasible in a particular comparison.

**FIGURE 6:**
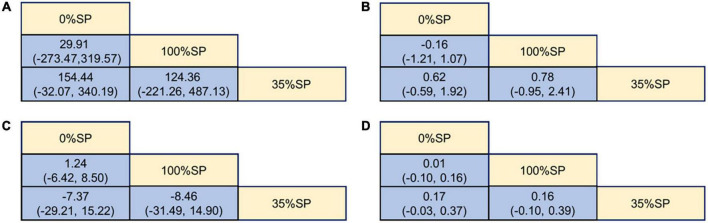
League Table of Kidney Indicators: **(A)** 24-h urine total protein (24hUTP), **(B)** Blood urea nitrogen (BUN), **(C)** Glomerular filtration rate (GFR), **(D)** Serum creatinine (Scr).

Based on the results of the network meta-analysis, we created an evidence network (Figure 5) and a league table (Figure 6). The effects of three different percentages of SP on four renal indices were demonstrated. The results indicate that for BUN, the mean difference between the 35% and 0% SP groups was −0.62 (95% CI −1.92, 0.59), and for Scr, it was −0.17 (95% CI −0.37, 0.03). While these findings did not reach statistical significance, there was an observable trend suggesting that the 35% SP diet performed better than the 0% SP diet. To further validate these trends, we conducted SUCRA ranking analysis.

#### 3.3.3. SUCRA ranking

**FIGURE 7:**
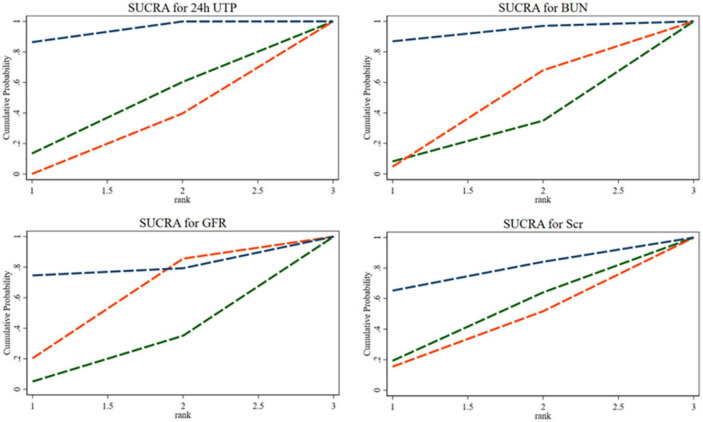
The bars are sorted by probability, representing their effectiveness in improving 24-h urine total protein (24hUTP), blood urea nitrogen (BUN), glomerular filtration rate (GFR), and serum creatinine (Scr). The green dashed line corresponds to the 100% SP diet, the red dashed line represents the 0% SP diet, and the blue dashed line represents the 35% SP diet.

The SUCRA ranking indicated that the 35% SP diet is the most beneficial among the three SP diet percentages for reducing 24hUTP and Scr. The probability rank was as follows: 35% SP > 100% SP > 0% SP. Similarly, for reducing BUN, as well as increasing GFR, the dietary composition with 35% SP was found to be the most advantageous, with the probability rank as follows: 35% SP > 0% SP > 100% SP. The probability-ranking diagram is illustrated in Figure 7. This ranking was consistent with the results of the network meta-analysis.

#### 3.3.4. Publication bias assessment

Despite the sample size being less than 10, we conducted a bias check for the study. Comparison-specific funnel charts showed that the studies were generally distributed in the upper middle section, suggesting a lower risk. However, individual studies appeared scattered at the bottom, which could be attributed to their small sample sizes (Supplementary Figure 2.1).

### 3.4. Effects of different percentages of soy protein on blood lipids

#### 3.4.1. Traditional pairwise and network meta-analyses

**FIGURE 8:**
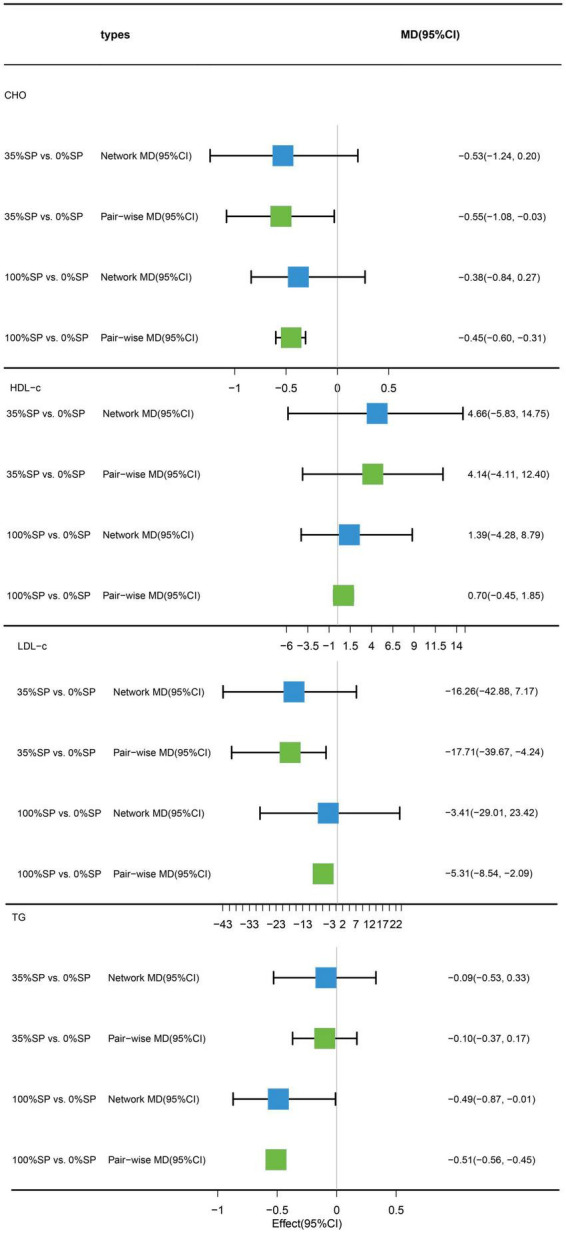
Network meta-analysis results (blue panels) and traditional pairwise meta-analysis results (green panels) show cholesterol (CHO), high-density lipoprotein cholesterol (HDL-C), low-density lipoprotein cholesterol (LDL-C), and triglycerides (TG) results. The line with the 95% CI represents the 95% CI in the network meta-analysis and the 95% CI in the traditional pairwise meta-analysis. When traditional meta-analyses are not feasible in specific comparisons, the results of a single trial can serve as direct evidence.

Traditional pairwise and network meta-analyses were performed on these four indicators. Results demonstrated that the efficacy of the 35% SP diet was higher than that of the 0% SP diet. However, the effects of CHO and LDL-C were statistically significant. Specifically, for the comparison between the 35% SP and 0% SP diets, the MD was −0.55 (95% CI: −1.08, −0.03) for CHO, and for LDL-C, it was −17.71 (95% confidence interval: −39.67, −4.24) (Figure 8).

#### 3.4.2. Network meta-analysis

**FIGURE 9:**
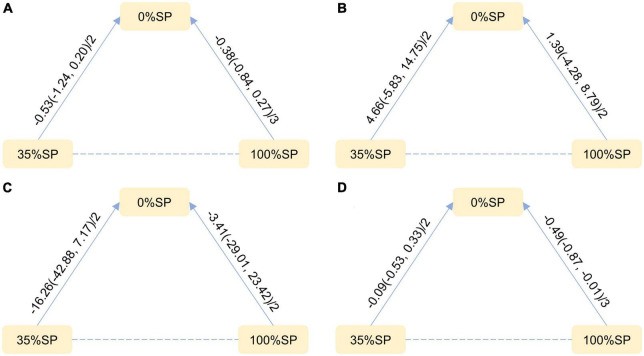
Network Evidence Map of blood lipid index: **(A)** Cholesterol (CHO), **(B)** High-density lipoprotein cholesterol (HDL-C), **(C)** Low-density lipoprotein cholesterol (LDL-C), **(D)** Triglycerides (TG). For pairwise comparisons in each indicator, arrows point to the less effective diet group, solid lines represent direct comparisons, and dashed lines represent indirect comparisons. Results for direct comparisons were network random effects meta-analyses expressed as MD (95% CI)/number of trials. Results from a single trial were provided when the meta-analysis was not feasible in a particular comparison.

**FIGURE 10:**
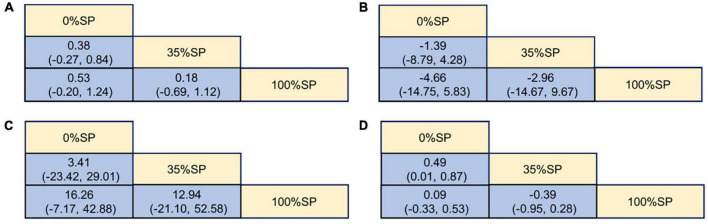
League Table of blood lipid index: **(A)** Cholesterol (CHO), **(B)** High-density lipoprotein cholesterol (HDL-C), **(C)** Low-density lipoprotein cholesterol (LDL-C), **(D)** Triglycerides (TG).

Based on the results of the network meta-analysis, we generated an evidence network (Figure 9) and a corresponding league table (Figure 10). These representations illustrated the effects of three different percentages of SP on four blood lipid indices. Specifically, for the comparison between 35% and 100% SP diets, the mean difference was 0.18 (95% CI −0.69, 1.12) for CHO, and −0.39 (95% CI −0.95, 0.28) for LDL-C. Although these results are not statistically significant, a trend was observed suggesting that the 35% SP diet might be more favorable than the 100% SP diet; therefore, we conducted a SUCRA ranking to verify this.

#### 3.4.3. SUCRA ranking

**FIGURE 11:**
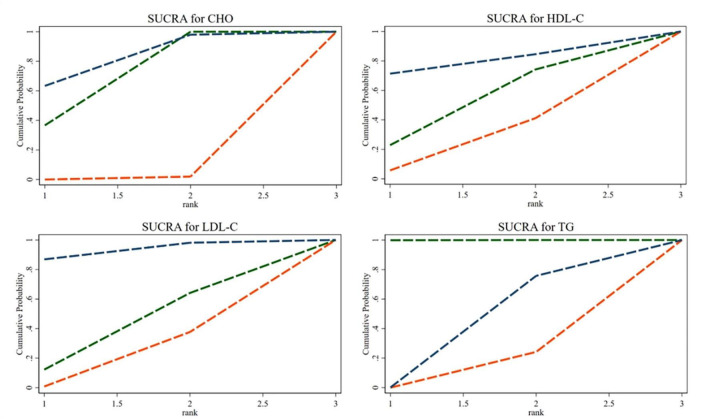
According to their effectiveness in improving cholesterol (CHO), high-density lipoprotein cholesterol (HDL-C), low-density lipoprotein cholesterol (LDL-C), and triglycerides (TG), they were sorted by probability. The green dashed line represents the 100% SP diet, the red dashed line means the 0% SP diet, and the blue dashed line represents the 35% SP diet.

The SUCRA ranking showed that CHO, HDL-C, LDL-C, and TG levels improved. The 35% SP diet was the most beneficial for CHO, HDL-C, and LDL-C. The probability ranking was 35% SP > 100% SP > 0% SP. The probability-ranking diagram is shown in Figure 11. This ranking was consistent with the results of the network meta-analysis.

#### 3.4.4. Publication bias assessment

Despite the sample size being less than 10, we conducted a bias check. The distribution of the comparison-specific funnel chart was acceptable but still suggested some potential publication bias (Supplementary Figure 2.2).

### 3.5. Effects of different percentages of soy protein on blood sugar and weight

#### 3.5.1. Traditional pairwise and network meta-analyses

**FIGURE 12:**
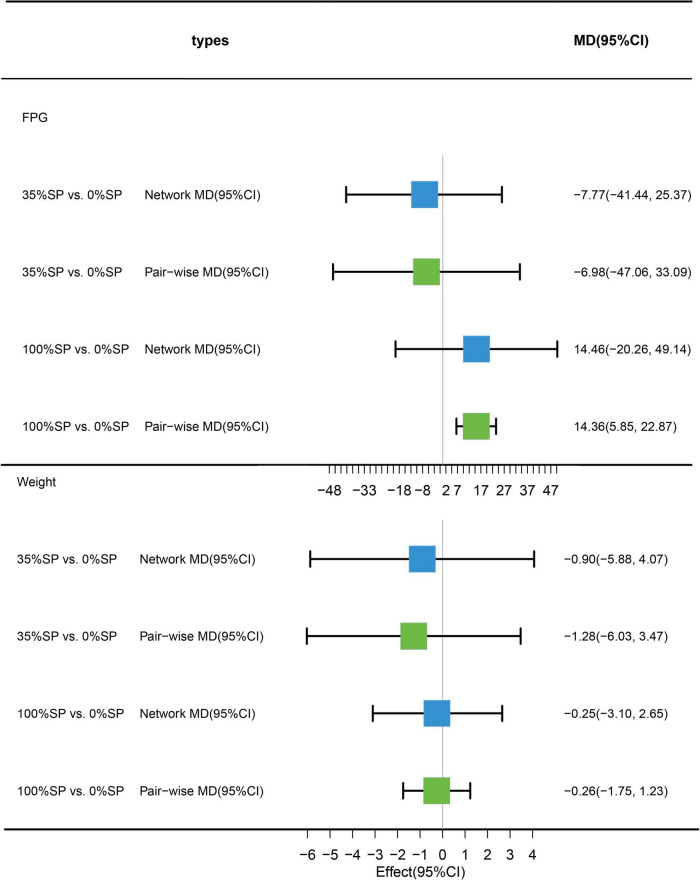
Network meta-analysis results (blue panels) and traditional pairwise meta-analysis (green panels) show results for FPG and weight. The line with the 95% CI represents the 95% CI in the network and traditional pairwise meta-analyses. When traditional meta-analyses are not feasible in specific comparisons, the results of a single trial can serve as direct evidence.

Traditional pairwise and network meta-analyses were performed on these three indicators. The results showed no statistical significance for FPG or weight, and the efficacy of the 35% SP diet was higher than that of the 0% SP diet (Figure 12).

#### 3.5.2. Network meta-analysis

**FIGURE 13:**
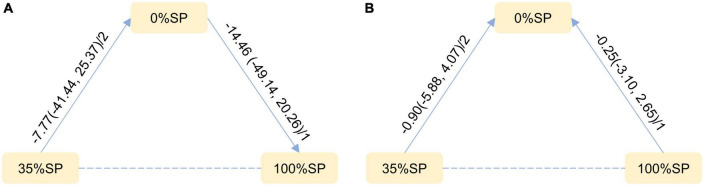
Network Evidence Map of blood glucose index: **(A)** Fasting blood glucose (FPG), **(B)** weight. For pairwise comparisons in each indicator, arrows point to the less effective diet group, solid lines represent direct comparisons, and dashed lines represent indirect comparisons. Results for direct comparisons were network random effects meta-analyses expressed as MD (95% CI)/number of trials. Results from a single trial were provided when the meta-analysis was not feasible in a particular comparison.

**FIGURE 14:**
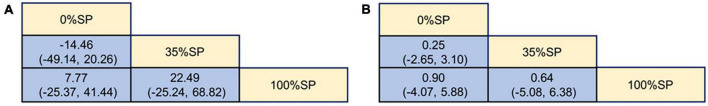
League Table of blood glucose index: **(A)** Fasting blood glucose (FPG), **(B)** weight.

Based on the results of the network meta-analysis, we generated an evidence network (Figure 13) and a corresponding league table (Figure 14). The effects of three different percentages of SP on FPG and body weight are shown. The results indicate that for comparison between 35% and 0% SP diets, the mean difference was −7.77 (95% CI −41.44, 25.37) for FPG, and −0.90 (95% CI −5.88, 4.07) for weight. These results were not statistically significant, and the 35% dominance trend was not obvious.

#### 3.5.3. SUCRA ranking

**FIGURE 15:**
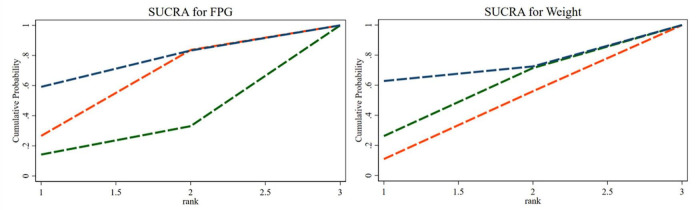
The bars are sorted by probability, representing their effectiveness in improving fasting blood glucose (FPG) and weight. The green dashed line corresponds to the 100% SP diet, the red dashed line represents the 0% SP diet, and the blue dashed line represents the 35% SP diet.

The SUCRA ranking indicated that, among the three percentages of SP diets, the 35% SP diet was the most beneficial for reducing FPG and weight. For FPG, the probability rank was 35% SP > 0% SP > 100% SP, while for weight, it was 35% SP > 100% SP > 0% SP. The probability-ranking diagram is illustrated in Figure 15.

#### 3.5.4. Publication bias assessment

Despite the sample size being less than 10, we conducted a bias check. The distribution of the comparison-specific funnel chart was acceptable but still suggested some potential publication bias (Supplementary Figure 2.3).

## 4. Discussion

Soy protein is a plant protein. Recently, the importance of plant-based diets for health has been increasingly recognized, especially in patients with kidney diseases. A meta-analysis by Aycart et al. (15) showed that for non-dialysis and dialysis patients with chronic kidney disease (CKD), eating plant proteins can reduce the inflammatory response compared with animal proteins. Based on the National Health and Nutrition Examination Survey (NHANES) database, the relationship between plant protein intake and mortality in patients with CKD was explored. The results showed that for populations with an estimated glomerular filtration rate of <60 ml/min/m^2^, increasing plant protein intake in the diet was associated with a decrease in all-cause mortality with the same total protein intake (16). According to the digestible essential amino acid score (DIAAS) method recommended by the Food and Agriculture Organization of the United Nations (FAO), Herreman et al. (17) calculated the DIAAS of 5 animal and 12 plant proteins. The results demonstrated that the SP score for people over 3 years old was >100, second only to whey.

Epidemiological and animal studies have demonstrated that endogenous estrogen in the human body has a protective effect on the kidneys (18). Soy isoflavones (SI) are the main active constituents of soy. Daidzein and Genistein are present in free and combined forms and account for 30 and 60% of the total SI, respectively. Studies have shown that SI and estrogen have similar structures and functions (19). Abnormal lipid metabolism in DN is associated with a decline in renal function (20). A meta-analysis by Wang et al. (21) showed that SI could improve DN by significantly reducing 24hUTP, BUN, FPG, blood lipids, and related inflammatory indicators. Another meta-analysis by Zhang et al. (22) showed that soy protein intake has a protective effect on Scr and serum phosphorus in patients with CKD and can significantly reduce serum TG concentration. Li et al. (23) administered different doses of SI to rats with metabolic syndrome by gavage for 4 weeks. The results showed that SI reduced the blood glucose levels and insulin resistance index of the rats compared to the model group. In a 4-week randomized crossover control trial, it was demonstrated that consumption of soymilk significantly reduced the systolic blood pressure in patients with T2DN in comparison to drinking milk (24). In conclusion, the potential mechanism of SP in reducing DN may be related to factors such as improving renal function, improving blood lipid levels, reducing blood glucose, and lowering blood pressure. The main component responsible for these beneficial effects appears to be SI.

Considering the beneficial effects of SP in patients with kidney disease, along with relevant studies, it appears that the extent of this effect might be related to different percentages of SP intake. Hence, the question arises whether T2DN patients can attain the most beneficial SP intake. To address this, we conducted a network meta-analysis to investigate the impact of different percentages of SP intake on patients with T2DN. Our analysis aimed to determine whether three different SP dietary protein percentages yield the same effect on the main renal outcome indicators and markers associated with glucose and lipid metabolism. Globally, ESRD caused by DN progression accounts for 30–50% of cases (25); it has an irreversible impact on the lives of sick people and places a heavy burden on public health. Therefore, we focused on renal function. In terms of renal function indicators, GFR is used to identify and stage DN (20). In addition, 24hUTP and Scr are important markers of renal function, and BUN is a biochemical parameter of renal function. These indicators were included in the analyses. We found that the 35% SP diet was the most effective in reducing Scr, BUN, 24hUTP, and increasing GFR among the three SP diets, which was better than the 0% SP and 100% SP diets; 24hUTP was statistically significant. CHO, TG, HDL-C, and LDL-C levels were selected for the analysis of blood lipid indicators. The results showed that, except for TG, 35% SP was the most beneficial among the three SP ratios for CHO, HDL-C, and LDL-C; CHO and LDL-C were statistically significant. The incidence of DN in obese patients with type 2 diabetes mellitus is >40% (26). We focused on weight and FPG levels. FPG levels and weight were not statistically significant. Additionally, the 35% SP diet was the best choice for improving blood sugar levels and weight.

The advantage of our study is that, before reporting more evidence of direct comparison, our network meta-analysis provides a comprehensive comparison of the prognosis of kidney, blood lipid, blood glucose, and weight of T2DN patients with different percentages of SP in their diet, which includes both direct and indirect comparisons. Not only can we compare the difference between the intake and non-intake of SP, but more importantly, we also analyzed the different percentages of SP in the diet. Particularly, for the 35% SP diet, most indicators showed the most positive effect on prognosis, and the results of the practical estimation were robust. By contrast, the sensitivity analysis results show little change, which is consistent with our initial assumption. The diet structure includes three proteins: SP, plant protein, and animal protein, the percentages of which are relatively balanced.

## 5. Limitations

This study has several limitations. First, owing to the small number of studies that met the inclusion criteria, it was difficult to unify the duration of the intervention. As a result, the most prolonged duration of the included studies was 4 years, and the shortest was only 4 weeks; this may be a significant source of heterogeneity. Fortunately, only one study lasted for more than 3 months. Second, dietary protein has two dimensions: quality and quantity. Strictly speaking, the amount should be controlled and consistent when studying quality; however, this is because of the limitations of the original research quantity. In addition, we did not standardize the amount of protein, which may have caused heterogeneity. However, by manually analyzing the results with heterogeneity and looking at possible sources of heterogeneity, it appeared that the difference in total protein intake was not the source of heterogeneity. This indicates that the proposed scheme is feasible. Third, patients with different dietary plans were at different DN stages, but an insufficient number of patients was unsuitable for any specific analysis. As a result, we only evaluated the effect of varying SP ratios on T2DN patients and were unable to explore different stages of kidney disease. Nevertheless, we included patients in the non-renal failure stage when formulating the inclusion criteria, which reduced the deviation of the results at various stages of kidney disease. Fourth, some studies did not clearly describe whether random sequence generation, allocation hiding, or blind methods were conducted, which may have affected the analysis results. However, we evaluated the results according to the ROB and the results were acceptable. Finally, patient characteristics and method quality, such as age, sex, duration of diabetes and nephropathy, blood glucose control level, and different randomization methods of the trial, may have been potentially uncontrollable factors in our analysis. However, in the network meta-analysis, whether differences in the characteristics of these patients have a substantial impact on the indicator results warrants further study.

## 6. Conclusion

Our analysis showed that for T2DN patients, different percentages of SP in the diet have other effects on major renal outcome indicators and markers related to glucose and lipid metabolism. When SP constitutes 35% of the total protein in the diet, it exhibits the most significant benefits in terms of Scr, BUN, 24hUTP, GFR, CHO, HDL-C, LDL-C, FPG, and weight. However, there are other more effective dietary protein structure percentages as well as long-term effectiveness, safety, and patient compliance. It is necessary to strictly design randomized controlled trials (RCTs) with multiple SP percentages, large sample sizes, and longer follow-up to confirm their impact on DN.

## Data availability statement

The original contributions presented in this study are included in the article/Supplementary material, further inquiries can be directed to the corresponding authors.

## Author contributions

LHZ, LZ, JS, and YW designed research methods. JS and RM collected and analyzed the data. JS, YW, and XZ wrote the original draft. LHZ, LZ, and BZ reviewed and edited the manuscript. All authors read and approved the final manuscript.

## References

[1] International Diabetes Federation. *IDF Diabetes Atlas. International Diabetes Federation.* 10th ed. Brussels: International Diabetes Federation (2021).

[2] Atkins RC Zimmet P. Diabetes: diabetic kidney disease: act now or pay later. *Nat Rev Nephrol.* (2010) 6:134–6. 10.1038/nrneph.2010.10 20186229

[3] Kanwar YS Sun L Xie P Liu F Chen S. A glimpse of various pathogenetic mechanisms of diabetic nephropathy. *Annu Rev Pathol.* (2011) 6:395–423. 10.1146/annurev.pathol.4.110807.092150 21261520 PMC3700379

[4] Oshima M Shimizu M Yamanouchi M Toyama T Hara A Furuichi K Trajectories of kidney function in diabetes: a clinicopathological update. *Nat Rev Nephrol.* (2021) 17:740–50. 10.1038/s41581-021-00462-y 34363037

[5] Saran R Robinson B Abbott KC Bragg-Gresham J Chen X Gipson D US renal data system 2019 Annual data report: epidemiology of kidney disease in the United States. *Am J Kidney Dis.* (2020) 75(Suppl1. 1):A6–7. 10.1053/j.ajkd.2019.09.003 31704083

[6] Lytvyn Y Bjornstad P Raalte DH Heerspink HL Cherney DZ. The new biology of diabetic kidney disease-mechanisms and therapeutic implications. *Endocr Rev.* (2020) 41:202–31. 10.1210/endrev/bnz010 31633153 PMC7156849

[7] Kramer H. Diet and chronic kidney disease. *Adv Nutr.* (2019) 10:S367–79. 10.1093/advances/nmz011 31728497 PMC6855949

[8] Alicic RZ Rooney MT Tuttle KR. Diabetic kidney disease: challenges, progress, and possibilities. *Clin J Am Soc Nephrol.* (2017) 12:2032–45. 10.2215/CJN.11491116 28522654 PMC5718284

[9] Zhang J Li J Huang JQ. Network meta-analysis of four Chinese patent medicines combined with angiotensin converting enzyme inhibitors or angiotensin receptor blockers in early diabetic nephropathy treatment. *World J Tradit Chin Med.* (2020) 6:51–60. 10.4103/wjtcm.wjtcm_41_19

[10] Brenner BM Meyer TW Hostetter TH. Dietary protein intake and the progressive nature of kidney disease: the role of hemodynamically mediated glomerular injury in the pathogenesis of progressive glomerular sclerosis in aging, renal ablation, and intrinsic renal disease. *N Engl J Med.* (1982) 307:652–9. 10.1056/NEJM198209093071104 7050706

[11] Anderson JW Blake JE Turner J Smith B. Effects of soy protein on renal function and proteinuria in patients with type 2 diabetes. *Am J Clin Nutr.* (1998) 68:1347S–53S. 10.1093/ajcn/68.6.1347S 9848497

[12] Lew QJ Jafar TH Koh HW Jin A Chow KY Yuan J Red meat intake and risk of ESRD. *J Am Soc Nephrol.* (2017) 28:304–12. 10.1681/ASN.2016030248 27416946 PMC5198288

[13] Page MJ McKenzie JE Bossuyt PM Boutron I Hoffmann T Mulrow C The PRISMA 2020 statement: an updated guideline for reporting systematic reviews. *BMJ.* (2021) 372:n71. 10.1136/bmj.n71 33782057 PMC8005924

[14] Higgins JPT Altman DG Gøtzsche PC Jüni P Moher D Oxman A The cochrane collaboration’s tool for assessing risk of bias in randomised trials. *BMJ.* (2011) 343:d5928. 10.1136/bmj.d5928 22008217 PMC3196245

[15] Aycart DF Acevedo S Eguiguren-Jimenez L Andrade J. Influence of plant and animal proteins on inflammation markers among adults with chronic kidney disease: a systematic review and meta-analysis. *Nutrients.* (2021) 13:1660. 10.3390/nu13051660 34068841 PMC8153567

[16] Chen X Wei G Jalili T Metos J Giri A Cho ME The associations of plant protein intake with all-cause mortality in CKD. *Am J Kidney Dis.* (2016) 67:423–30. 10.1053/j.ajkd.2015.10.018 26687923 PMC4769135

[17] Herreman L Nommensen P Pennings B Laus MC. Comprehensive overview of the quality of plant- and animal-sourced proteins based on the digestible indispensable amino acid score. *Food Sci Nutr.* (2020) 8:5379–91. 10.1002/fsn3.1809 33133540 PMC7590266

[18] Garovic VD August P. Sex differences and renal protection: keeping in touch with your feminine side. *J Am Soc Nephrol.* (2016) 27:2921–4. 10.1681/ASN.2016040454 27188841 PMC5042684

[19] Hu C Wong W Wu R Lai W. Biochemistry and use of soybean isoflavones in functional food development. *Crit Rev Food Sci Nutr.* (2020) 60:2098–112. 10.1080/10408398.2019.1630598 31272191

[20] Tuttle KR Bakris GL Bilous RW Chiang JL Boer IH Goldstein-Fuchs J Diabetic kidney disease: a report from an ADA Consensus Conference. *Diabetes Care.* (2014) 37:2864–83. 10.2337/dc14-1296 25249672 PMC4170131

[21] Wang X Liang Q Zeng X Huang G Xin G Xu Y Effects of soy isoflavone supplementation on patients with diabetic nephropathy: a systematic review and meta-analysis of randomized controlled trials. *Food Funct.* (2021) 12:7607–18. 10.1039/d1fo01175h 34236368

[22] Zhang J Liu J Su J Tian F. The effects of soy protein on chronic kidney disease: a meta-analysis of randomized controlled trials. *Eur J Clin Nutr.* (2014) 68:987–93. 24939439 10.1038/ejcn.2014.112

[23] Li L Ye L Fengrui X. Effects of soybean Isoflavone on glucose and lipid metabolism, serum leptin and FFA in rats with diet induced metabolic syndrome. *J Tradit Chin Med.* (2012) 40:85–7.

[24] Miraghajani MS Najafabadi MM Surkan PJ Esmaillzadeh A Mirlohi M Azadbakht L. Soy milk consumption and blood pressure among type 2 diabetic patients with nephropathy. *J Renal Nutr.* (2013) 23:277–82. 10.1053/j.jrn.2013.01.017 23498346

[25] Ruiz-Ortega M Rodrigues-Diez RR Lavoz C Rayego-Mateos S. Special Issue “Diabetic nephropathy: diagnosis, prevention and treatment”. *J Clin Med.* (2020) 9:813. 10.3390/jcm9030813 32192024 PMC7141346

[26] Docherty NG Canney AL Le Roux CW. Weight loss interventions and progression of diabetic kidney disease. *Curr Diabetes Rep.* (2015) 15:55. 10.1007/s11892-015-0625-2 26122095

[27] Azadbakht L Shakerhosseini R Atabak S Jamshidian M Mehrabi Y Esmaill-Zadeh A. Beneficiary effect of dietary soy protein on lowering plasma levels of lipid and improving kidney function in type II diabetes with nephropathy. *Eur J Clin Nutr.* (2003) 57:1292–4. 10.1038/sj.ejcn.1601688 14506491

[28] Teixeira SR Tappenden KA Carson L Jones R Prabhudesai M Marshall WP Isolated soy protein consumption reduces urinary albumin excretion and improves the serum lipid profile in men with type 2 diabetes mellitus and nephropathy. *J Nutr.* (2004) 134:1874–80. 10.1093/jn/134.8.1874 15284369

[29] Azadbakht L Atabak S Esmaillzadeh A. Soy protein intake, cardiorenal indices, and C-reactive protein in type 2 diabetes with nephropathy. *Diabetes Care.* (2008) 31:648–54. 10.2337/dc07-2065 18184902

[30] Azadbakht L Esmaillzadeh A. Soy-protein consumption and kidney-related biomarkers among type 2 diabetics: a crossover randomized clinical trial. *J Renal Nutr.* (2009) 19:479–86. 10.1053/j.jrn.2009.06.002 19758824

