# Supplementary material for: Comparison of the effects of different percentages of soy protein in the diet on patients with type 2 diabetic nephropathy: systematic reviews and network meta-analysis
Source: Front Nutr. 2023 Aug 24;10:1184337. doi: 10.3389/fnut.2023.1184337 (PMC10484530; doi:10.3389/fnut.2023.1184337)
Supplement: Supplementary file 1 [file Data_Sheet_1.docx]

**Comparison of the effects of different proportions of soy protein in the diet on patients with type 2 Diabetic Nephropathies: Systematic reviews and network meta-analysis**

Jun Sun et al.

Online Supplemental Material

**Supplemental Table 1** Search strategy

| **Database** | **Search strategy** | | |
| --- | --- | --- | --- |
| **Pubmed** | (((((((((((((((((((Diabetic Nephropathies[MeSH Terms]) OR (Nephropathies, Diabetic[Title/Abstract])) OR (Nephropathy, Diabetic[Title/Abstract])) OR (Diabetic Nephropathy[Title/Abstract])) OR (Diabetic Kidney Disease[Title/Abstract])) OR (Diabetic Kidney Diseases[Title/Abstract])) OR (Kidney Disease, Diabetic[Title/Abstract])) OR (Kidney Diseases, Diabetic[Title/Abstract])) OR (Diabetic Glomerulosclerosis[Title/Abstract])) OR (Glomerulosclerosis, Diabetic[Title/Abstract])) OR (Intracapillary Glomerulosclerosis[Title/Abstract])) OR (Nodular Glomerulosclerosis[Title/Abstract])) OR (Glomerulosclerosis, Nodular[Title/Abstract])) OR (Kimmelstiel-Wilson Syndrome[Title/Abstract])) OR (Kimmelstiel Wilson Syndrome[Title/Abstract])) OR (Syndrome, Kimmelstiel-Wilson[Title/Abstract])) OR (Kimmelstiel-Wilson Disease[Title/Abstract])) OR (Kimmelstiel Wilson Disease[Title/Abstract])) AND (((((((((((((((((Soybean Proteins[MeSH Terms]) OR (Soy Bean Proteins[Title/Abstract])) OR (Soybean Protein[Title/Abstract])) OR (Protein, Soybean[Title/Abstract])) OR (Soy Bean Protein[Title/Abstract])) OR (Bean Protein, Soy[Title/Abstract])) OR (Protein, Soy Bean[Title/Abstract])) OR (Dietary Soybean Proteins[Title/Abstract])) OR (Dietary Soybean Protein[Title/Abstract])) OR (Protein, Dietary Soybean[Title/Abstract])) OR (Proteins, Dietary Soybean[Title/Abstract])) OR (Soybean Protein, Dietary[Title/Abstract])) OR (Soybean Proteins, Dietary[Title/Abstract])) OR (Soy Proteins[Title/Abstract])) OR (Protein, Soy[Title/Abstract])) OR (Proteins, Soy[Title/Abstract])) OR (Soy Protein[Title/Abstract]))) AND (randomized controlled trial[Publication Type] OR randomized[Title/Abstract] OR placebo[Title/Abstract]) | | |
| **Embase** | ('diabetic nephropathy'/exp OR 'diabetic nephropathy':ab,ti OR 'nephropathies, diabetic':ab,ti OR 'nephropathy, diabetic':ab,ti OR 'diabetic nephropathies':ab,ti OR 'diabetic kidney disease':ab,ti OR 'diabetic kidney diseases':ab,ti OR 'kidney disease, diabetic':ab,ti OR 'kidney diseases, diabetic':ab,ti OR 'diabetic glomerulosclerosis':ab,ti OR 'glomerulosclerosis, diabetic':ab,ti OR 'intracapillary glomerulosclerosis':ab,ti OR 'nodular glomerulosclerosis':ab,ti OR 'glomerulosclerosis, nodular':ab,ti OR 'kimmelstiel-wilson syndrome':ab,ti OR 'kimmelstiel wilson syndrome':ab,ti OR 'syndrome, kimmelstiel-wilson':ab,ti OR 'kimmelstiel-wilson disease':ab,ti OR 'kimmelstiel wilson disease':ab,ti) AND ('soybean protein'/exp OR 'soybean proteins':ab,ti OR 'soy bean proteins':ab,ti OR 'soybean protein':ab,ti OR 'protein, soybean':ab,ti OR 'soy bean protein':ab,ti OR 'bean protein, soy':ab,ti OR 'protein, soy bean':ab,ti OR 'dietary soybean proteins':ab,ti OR 'dietary soybean protein':ab,ti OR 'protein, dietary soybean':ab,ti OR 'proteins, dietary soybean':ab,ti OR 'soybean protein, dietary':ab,ti OR 'soybean proteins, dietary':ab,ti OR 'protein, soy':ab,ti OR 'soy proteins':ab,ti OR 'proteins, soy':ab,ti OR 'soy protein':ab,ti) AND ('randomized controlled trial':ab,ti OR 'randomized':ab,ti OR 'placebo':ab,ti) | | |
| **Cochrane** | **#** | Terms |  |
|  | **1** | (Diabetic Nephropathy):ti,ab,kw OR (Nephropathies,Diabetic):ti,ab,kw OR (Nephropathy.Diabetic):ti,ab,kw OR (Diabetic Nephropathies):ti,ab,kw OR (Diabetic Kidney Disease):ti,ab,kw OR (Diabetic Kidney Diseases):ti,ab,kw OR (Kidney Disease.Diabetic):ti,ab,kw OR (Kidney Diseases,Diabetic):ti,ab,kw OR (Diabetic Glomerulosclerosis):ti.ab.kw OR (Glomerulosclerosis.Diabetic):ti.ab.kw OR (Intracapillary Glomerulosclerosis):ti.ab.kw OR (Nodular Glomerulosclerosis):ti.ab.kw OR (Glomerulosclerosis,Nodular):ti,ab,kw OR (Kimmelstiel-Wilson Syndrome):ti,ab,kw OR (Kimmelstiel Wilson Syndrome):ti,ab,kw OR (Syndrome,Kimmelstiel-Wilson):ti.ab,kw OR (Kimmelstiel-Wilson Disease):ti,ab,kw OR (Kimmelstiel Wilson Disease):ti,ab,kw |  |
|  | **2** | MeSH descriptor: [Diabetic Nephropathies] explode all trees |  |
|  | **3** | #1 OR #2 |  |
|  | **4** | MeSH descriptor: [Soybean Proteins] explode all trees |  |
|  | **5** | (Soybean Proteins):ti,ab,kw OR (Soy Bean Proteins):ti,ab,kw OR (Soybean Protein):ti,ab,kw OR (Protein,Soybean):ti,ab,kw OR (Soy Bean Protein):ti,ab,kw OR (Bean Protein,Soy):ti,ab,kw OR (Protein.Soy Bean):ti,ab,kw OR (Dietary Soybean Proteins):ti,ab,kw OR (Dietary Soybean Protein):ti.ab,kw OR (Protein.Dietary Soybean):ti,ab.kw OR (Proteins,Dietary Soybean):ti,ab.kw OR (Soybean Protein.Dietary):ti,ab.kw OR (Soybean Proteins.Dietary):ti,ab,kw OR (Soy Proteins):ti,ab.kw OR (Protein,Soy):ti,ab,kw OR (Proteins,Soy):ti,ab,kw OR (Soy Protein):ti,ab,kw |  |
|  | **6** | #4 OR #5 |  |
|  | **7** | #3 AND #6 |  |
|  | **8** | MeSH descriptor: [Soy Foods] explode all trees |  |
|  | **9** | (Soy Foods):ti,ab,kw OR (Food,Soy):ti.ab.kw OR (Foods.Soy):ti,ab,kw OR (Soy Food):ti.ab.kw OR (Natto):ti.ab,kw OR (Soy Cheese):ti,ab,kw OR (Cheese.Soy):ti,ab.kw OR (Cheeses,Soy):ti,ab,kw OR (Soy Cheeses):ti,ab,kw OR (Tempeh):ti,ab.kw OR (Texturized Soy Protein):ti.ab.kw OR (Protein,Texturized Soy):ti,ab,kw OR (ProteinsTexturized Soy):ti,ab,kw OR (Soy Protein.Texturized):ti,ab,kw OR (Soy Proteins,Texturized):ti,ab,kw OR (Texturized Soy Proteins):ti,ab.kw OR (Texturized Vegetable Protein):ti,ab,kw OR (Protein,Texturized Vegetable):ti,ab.kw OR (Vegetable Protein.Texturized):ti,ab,kw OR (Vegetable Proteins.Texturized):ti,ab,kw OR (Tofu):ti,ab,kw OR (Bean Curd,Soy):ti,ab,kw OR (Bean Curds,Soy):ti,ab,kw OR (Curd,Soy Bean):ti,ab,kw OR (Curds,Soy Bean):ti,ab,kw OR (Soy Bean Curd):ti,ab,kw OR (Soy Bean Curds):ti.ab.kw OR (Miso):ti.ab.kw OR (Soy Sauce):ti,ab,kw OR (Sauce,Soy):ti,ab.kw |  |
|  | **10** | #8 OR #9 |  |
|  | **11** | #3 AND #10 |  |
| **Web of science** | TS=(Diabetic Nephropathies OR Nephropathies, Diabetic OR Nephropathy, Diabetic OR Diabetic Nephropathy OR Diabetic Kidney Disease OR Diabetic Kidney Diseases OR Kidney Disease, Diabetic OR Kidney Diseases, Diabetic OR Diabetic Glomerulosclerosis OR Glomerulosclerosis, Diabetic OR Intracapillary Glomerulosclerosis OR Nodular Glomerulosclerosis OR Glomerulosclerosis, Nodular OR Kimmelstiel-Wilson Syndrome OR Kimmelstiel Wilson Syndrome OR Syndrome, Kimmelstiel-Wilson OR Kimmelstiel-Wilson Disease OR Kimmelstiel Wilson Disease) AND TS=(Soybean Proteins OR Soy Bean Proteins OR Soybean Protein OR Protein, Soybean OR Soy Bean Protein OR Bean Protein, Soy OR Protein, Soy Bean OR Dietary Soybean Proteins OR Dietary Soybean Protein OR Protein, Dietary Soybean OR Proteins, Dietary Soybean OR Soybean Protein, Dietary OR Soybean Proteins, Dietary OR Soy Proteins OR Protein, Soy OR Proteins, Soy OR Soy Protein) AND TS=(randomized controlled trial OR randomized OR placebo) | |  |
| **Clinical trails** | Condition or disease：“Diabetic nephropathy” OR “Diabetic kidney disease”  Study type：Interventional studies (clinical trials)  Study Results: All studies | |  |
| **CNKI** | (((subject=diabetic nephropathy or title=diabetic nephropathy or v_subject=Chinese-English extension (diabetic nephropathy) or title=Chinese-English extension (diabetic nephropathy)) or (subject=diabetic glomerulosclerosis or title=diabetic glomerulosclerosis or v_subject=Chinese-English extension (diabetic glomerulosclerosis) or title=diabetic glomerulosclerosis))) or ((subject=diabetic nephropathy or title=diabetic nephropathy or v_subject=diabetic nephropathy or title=diabetic nephropathy)) or (subject=diabetic nephrotic syndrome or title=diabetic nephrotic syndrome or v_ subject=diabetic nephropathy syndrome or title=diabetic nephropathy syndrome))) and ((subject=soy protein or title=soy protein or v_subject=soy protein or title=soy protein)) or (subject=soy isolate or title=soy isolate or v_subject=soy protein)) or (subject=soy isolate or title=soy protein or v_subject=soy protein)) Soy Protein or v_subject=Soy Protein or title=Soy Protein)) or ((Subject=Soy Diet or title=Soy Diet or v_subject=Soy Diet or title=Soy Diet)) or (Subject=Soy Isoflavones or title=Soy Isoflavones or v_subject=Soy Isoflavones or title=Soy Isoflavones)) ) ) and ( (abstract=Random Control) or (abstract=Random) ) or ( (abstract=Random Assignment) or (abstract=RCT) ) ) (fuzzy match) | |  |
| **CBM** | **#** | Terms |  |
|  | **1** | "Diabetic nephropathy" [unweighted:extended] |  |
|  | **2** | "Diabetic glomerulosclerosis" [common field:smart] OR "Diabetic nephrotic syndrome" [common field:smart] |  |
|  | **3** | (#2) OR (#1) |  |
|  | **4** | "Soy foods" [unweighted:extended] |  |
|  | **5** | "Soy protein group" [unweighted:extended] |  |
|  | **6** | "Soybean Isolated Protein"[Common Field:Smart] OR "Soy Isoflavones"[Common Field:Smart] OR "Soy Protein"[Common Field:Smart] OR "Soy Milk"[Common Field:Smart] OR "Tofu"[Common Field:Smart] OR "Beans"[Common Field:Smart] |  |
|  | **7** | "Soy Diet" [common field: Smart] |  |
|  | **8** | (#7) OR (#6) OR (#5) OR (#4) |  |
|  | **9** | "Randomized controlled trials" [unweighted:extended] |  |
|  | **10** | "Random Cross Reference" [Common Field: Smart] OR "Random Assignment" [Common Field: Smart] OR "Random" [Common Field: Smart] OR "RCT" [Common Field: Smart] |  |
|  | **11** | (#9) 0R (#10) |  |
|  | **12** | (#12) AND (#9) AND (#3) |  |
| **WanFang** | Subject:(diabetic nephropathy or diabetic glomerulosclerosis or diabetic nephrotic syndrome) and Subject:(soy protein or soy isolate or soy diet or soy isoflavones or soy milk or tofu or beans) and Subject:(randomized control or randomized or RCT or random assignment) | |  |
| **VIP** | (A=diabetic nephropathy or diabetic glomerulosclerosis or diabetic nephrotic syndrome) and (A=soy protein or soy isolate or soy diet or soy isoflavones or soy milk or tofu or beans) and (R=randomized control or randomized or RCT or random assignment) | |  |

**Supplemental Table 2** Results and data included in the study.


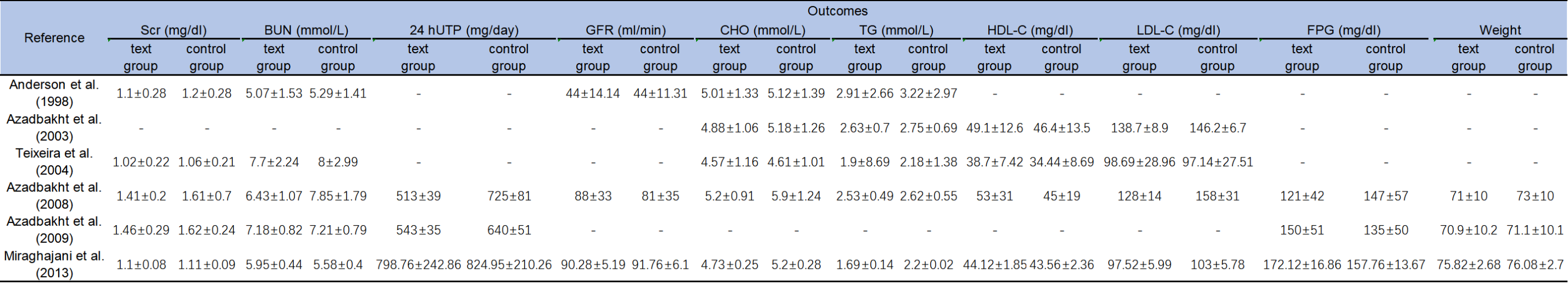


**Supplemental Figure 1** Sensitivity analysis

Sensitivity analysis

| Intervention | 24hUTP | |  | BUN | |  | GFR | |  | Scr | |
| --- | --- | --- | --- | --- | --- | --- | --- | --- | --- | --- | --- |
|  | heterogeneity | analysis |  | heterogeneity | analysis |  | heterogeneity | analysis |  | heterogeneity | analysis |
| 100%SP vs. 0%SP | NS | × |  | I²=0% | × |  | I²=0% | × |  | I²=0% | × |
| 35%SP vs. 0%SP | I²=95% | √ |  | I²=84% | √ |  | NS | × |  | I²=0% | √ |


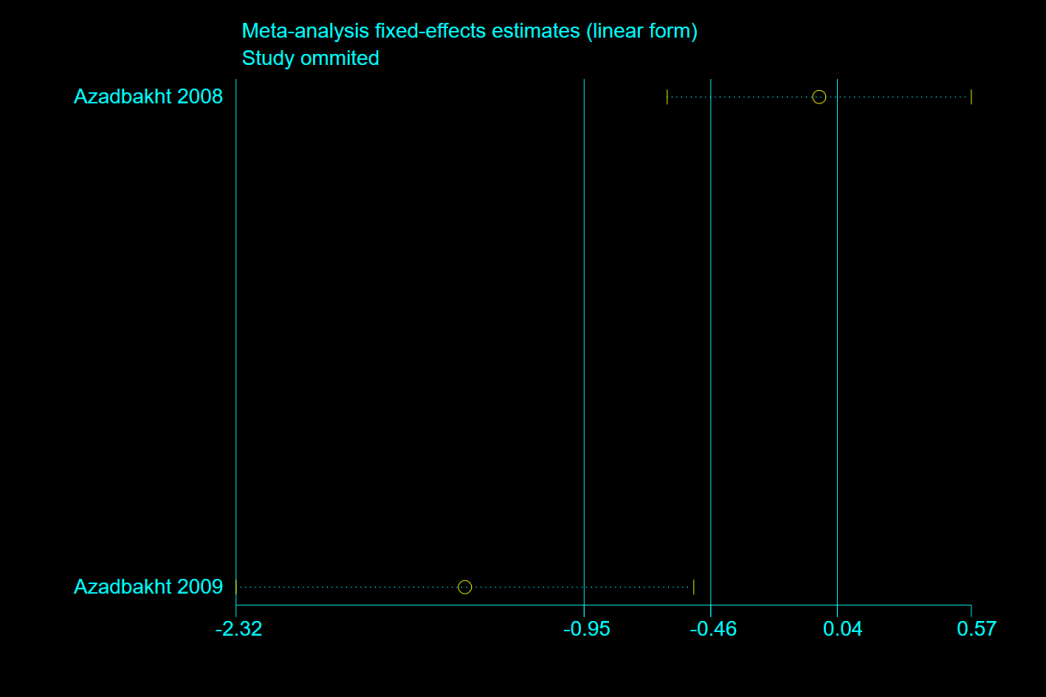


BUN


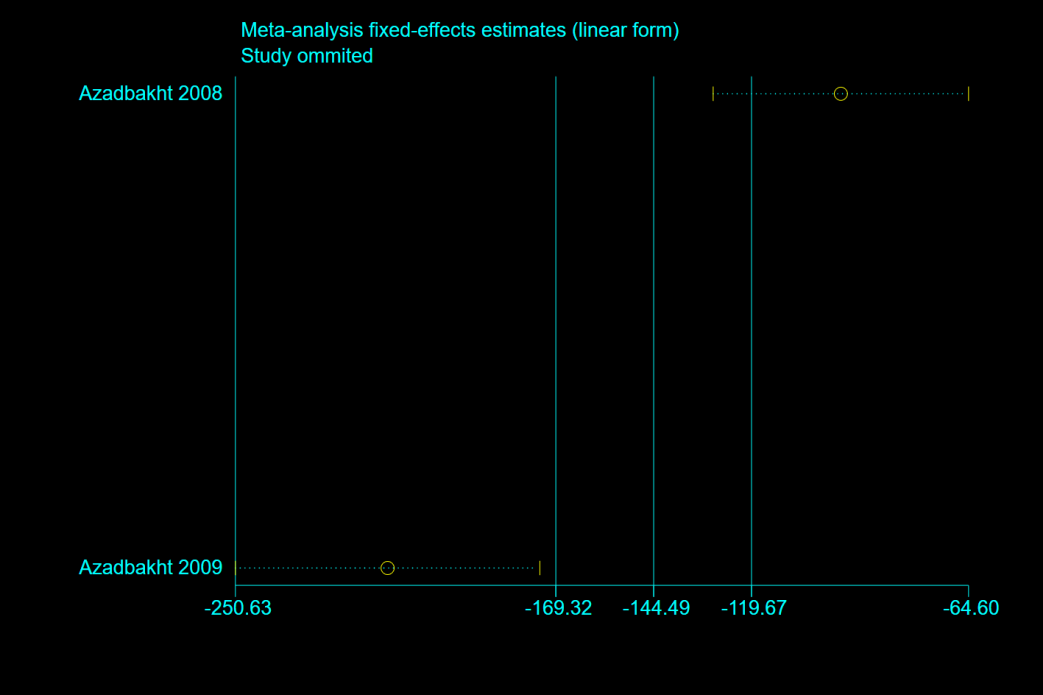


24hUTP

Sensitivity analysis

| Intervention | CHO | |  | HDL-C | |  | LDL-C | |  | TG | |
| --- | --- | --- | --- | --- | --- | --- | --- | --- | --- | --- | --- |
|  | heterogeneity | analysis |  | heterogeneity | analysis |  | heterogeneity | analysis |  | heterogeneity | analysis |
| 100%SP vs. 0%SP | I²=0% | × |  | I²=31% | × |  | I²=0% | × |  | I²=0% | × |
| 35%SP vs. 0%SP | I²=0% | √ |  | I²=0% | × |  | I²=87% | × |  | I²=0% | √ |


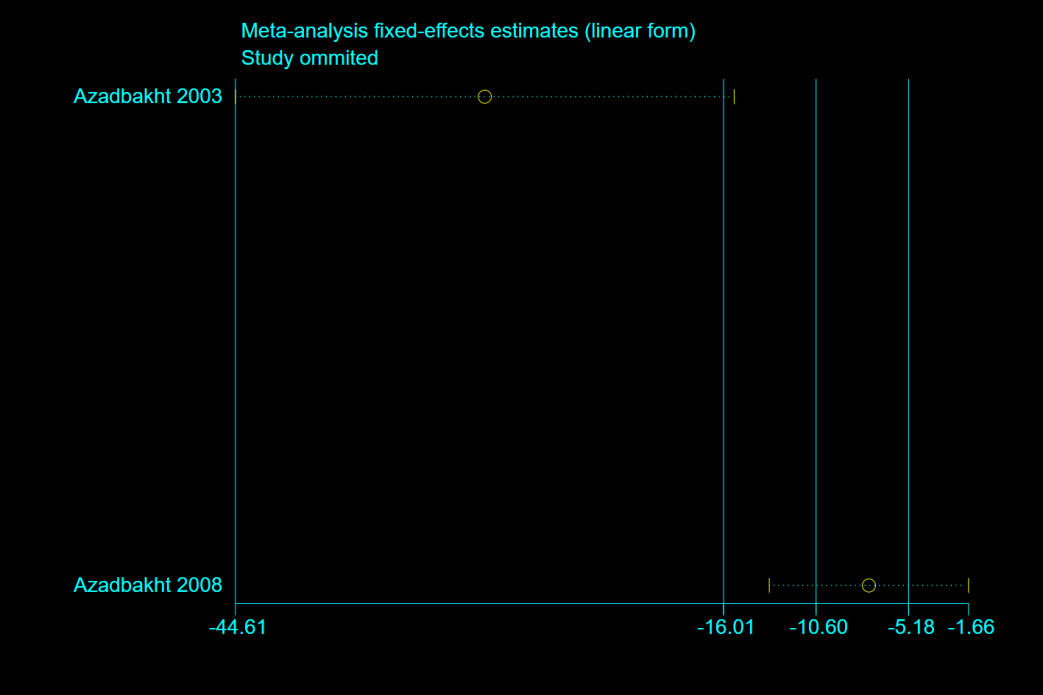


LDL-C

Sensitivity analysis

| Intervention | FPG | |  |  | Weight | |
| --- | --- | --- | --- | --- | --- | --- |
|  | heterogeneity | analysis |  |  | heterogeneity | analysis |
| 100%SP vs. 0%SP | NS | × |  |  | NS | × |
| 35%SP vs. 0%SP | I²=64% | √ |  |  | I²=0% | × |


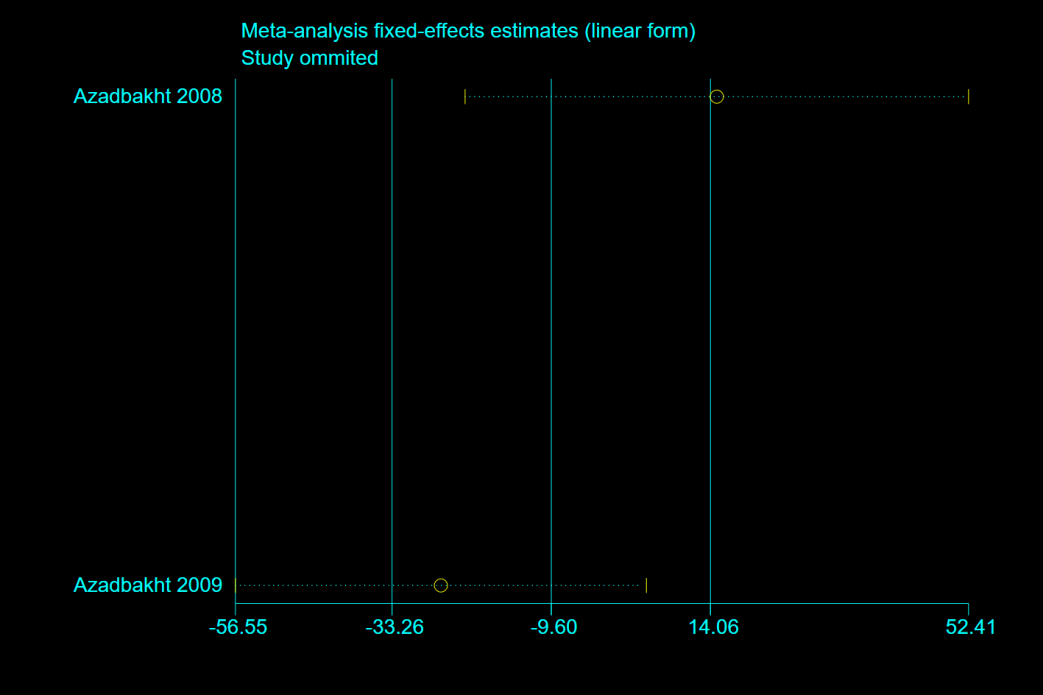


FPG

**Supplemental Figure 2** Publication bias assessment

**
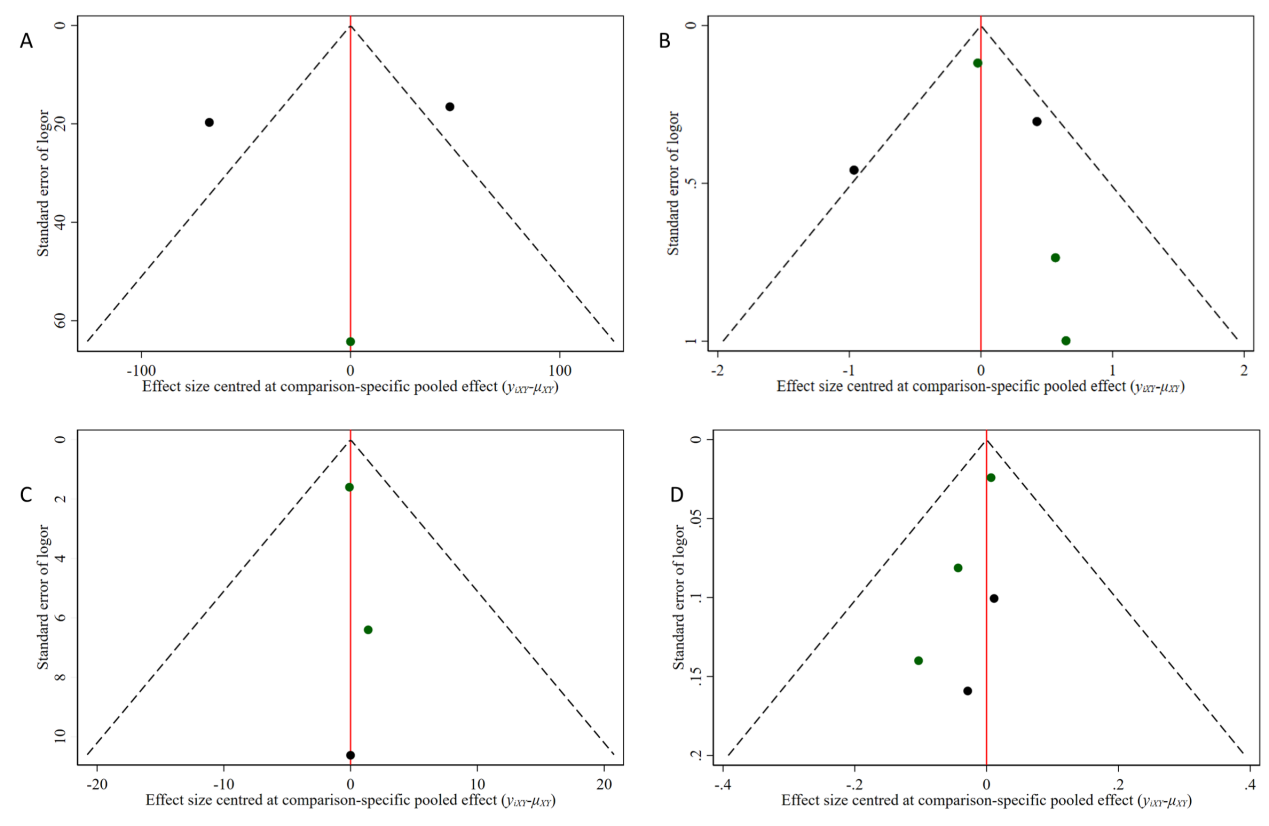
**

**FIGURE 2.1** Comparison-specific funnel chart in terms of (A) 24hUTP, (B) BUN, (C) GFR, (D) Scr. Green, 0%SP vs 100%SP; black, 100%SP vs 35%SP, for A and B; black, 0%SP vs 35%SP, for C and D.


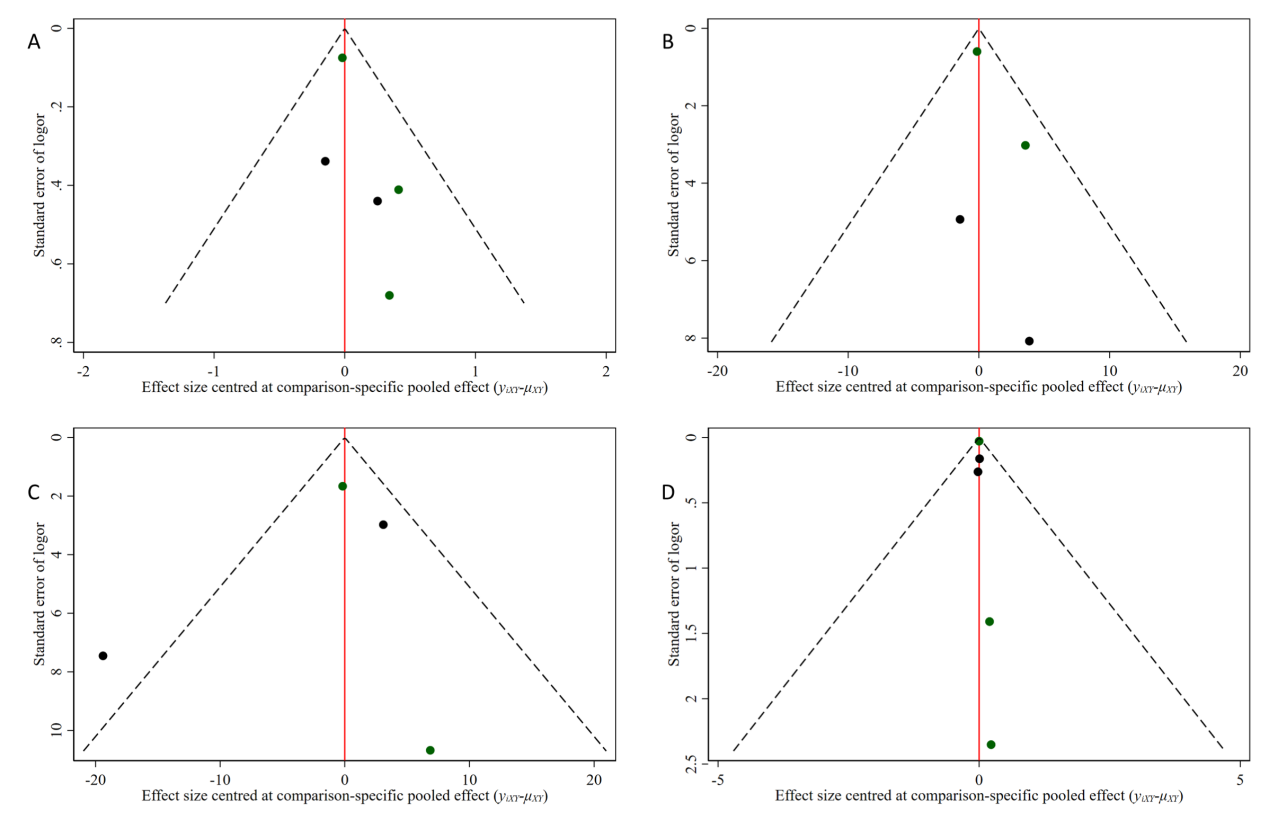


**FIGURE 2.2** Comparison-specific funnel chart in terms of (A) CHO, (B) HDL-C, (C) LDL-C, (D) TG. Green, 0%SP vs 100%SP; black, 0%SP vs 35%SP.

**FIGURE 2.3**
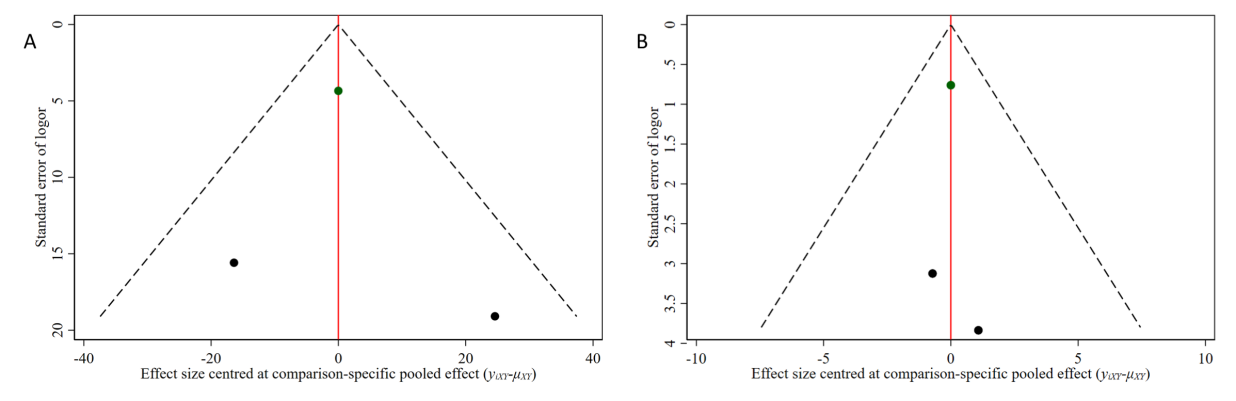
Comparison-specific funnel chart in terms of (A) FPG, (B) weight. Green, 0%SP vs 100%SP; black, 0%SP vs 35%SP, for A; black, 100%SP vs 35%SP, for B.
